# The Emerging Landscapes of Long Noncoding RNA in Thyroid Carcinoma: Biological Functions and Clinical Significance

**DOI:** 10.3389/fonc.2021.706011

**Published:** 2021-08-10

**Authors:** Jian Zhu, Changrui Liu, Dan Wang, Xianjiao Cao, Shuai Wang, Yixin Liu, Jun Wang, Peifeng Li, Qingqing He

**Affiliations:** ^1^The First School of Clinical Medicine, Shandong University of Traditional Chinese Medicine, Jinan, China; ^2^Department of Thyroid and Breast Surgery, The 960th Hospital of the Chinese People’s Liberation Army, Jinan, China; ^3^Department of Pathology, The 960th Hospital of the Chinese People’s Liberation Army, Jinan, China

**Keywords:** long noncoding RNA, thyroid carcinoma, biomarker, therapeutic target, clinical significance

## Abstract

Thyroid carcinoma (TC) is one of the most prevalent primary endocrine tumors, and its incidence is steadily and gradually increasing worldwide. Accumulating evidence has revealed the critical functions of long noncoding RNAs (lncRNAs) in the tumorigenesis and development of TC. Many TC-associated lncRNAs have been documented to be implicated in TC malignant behaviors, including abnormal cell proliferation, enhanced stem cell properties and aggressiveness, and resistance to therapeutics, through interaction with proteins, DNA, or RNA or encoding small peptides. Therefore, further elucidating the lncRNA dysregulation sheds additional insights into TC tumorigenesis and progression and opens new avenues for the early diagnosis and clinical therapy of TC. In this review, we summarize the abnormal expression of lncRNA in TC and the fundamental characteristics in TC tumorigenesis and development. Additionally, we introduce the potential prognostic and therapeutic significance of lncRNAs in TC.

## Introduction

Thyroid carcinoma (TC) is the most prevalent malignancy of the endocrine system and accounts for about 1% of all malignancies. The yearly incidence of TC in 2019 was 15.94 per 100,000 population (1). Currently, TC ranks the fourth most common cancer for women (2, 3). Early TC-associated investigation mainly focused on the biological functions of protein-coding genes (PCGs) for their fundamental functions in the regulation of signaling transduction and various biological activities. Therefore, elucidating the functional roles and molecular mechanisms of noncoding RNAs (ncRNAs) in TC tumorigenesis is of critical significance. It has been well accepted that less than 2% of genome sequence encodes proteins, whereas the remaining 98% genome sequences do not have coding potential and their transcriptional products (ncRNAs) have not been functionally characterized. Moreover, in the past, this was once considered to be “transcriptional noise” (4–6). Based on the length of transcripts, ncRNAs can be subdivided into small ncRNAs (<200 base pairs) or long ncRNAs (>200 base pairs, lncRNAs). The small ncRNAs that play crucial roles in tumorigenesis could be further classified as miRNAs, tsRNAs, and piRNAs. LncRNAs are transcripts like mRNA in length ranging from 200 nt to ~100 kilobases (kb) lacking significant open reading frames and have no coding potential. LncRNAs include subclasses such as pseudogenes and circRNAs (7–9). At present, human ncRNAs include more than 10,000 small ncRNA genes, approximately 14,500 pseudogenes, and almost 16,000 lncRNA genes according to the GENCODE Release 24 annotation (10). LncRNAs are characterized by extremely weak expression, worse conservation in different species, and specifically expressed in different tissues and developmental stages (11). Recently, a growing body of evidence suggests that ncRNAs, particularly lncRNA, have emerged as important regulators of gene expression during signaling transduction and diverse physiological and pathological processes through interacting with RNA, DNA, and protein and forming RNA–RNA, RNA–DNA, and RNA–protein complexes, thereby regulating gene expression *via* various molecular mechanisms, including modulation of transcription, mRNA stability, and translation (12, 13).

The first lncRNA was identified in fetal liver tissue in 1990 (14). The following year, X chromosome inactivation mediated by one of the most famous lncRNA XIST was found (15). To date, more than 50,000 genes have been found to transcribe lncRNA (16), and this number is still steadily and rapidly increasing. Emerging evidence has been well accepted that lncRNAs are involved in gene regulation at the transcriptional or posttranscriptional level and play critical roles in signaling transduction and multiple physiological and pathological disease initiation and processes. LncRNAs were characterized by differentially expressing different organ types and cancers including TC (17–19). Currently, the functional roles of lncRNAs in TC tumorigenesis and development remain largely unknown, and only a small part of lncRNAs has been extensively studied (3, 20). Interestingly, many lncRNAs were identified in body fluids, which were easily detected and analyzed, making them hold the potential to act as attractive biomarkers in liquid biopsy of TC. For example, lncRNA HOTAIR is overexpressed in the serum and is an independent prognostic marker to predict lymph node metastasis of papillary thyroid carcinoma (PTC) (21). The unique and pivotal characteristics of lncRNA reveal their attractive clinical significance in the TC diagnosis and targeted therapy. LncRNAs bear a tremendous potential to improve our understanding of the steps involved in TC tumorigenesis. In this review, we summarize the aberrant expression of lncRNA in TC and generally introduce the functional roles of lncRNA-mediated pathogenesis in TC tumorigenesis by functioning as tumor-suppressor gene or oncogene. Additionally, we discuss the prognostic, diagnostic, and therapeutic potentials of lncRNA in TC.

## Classification of Human TC

**Figure 1:**
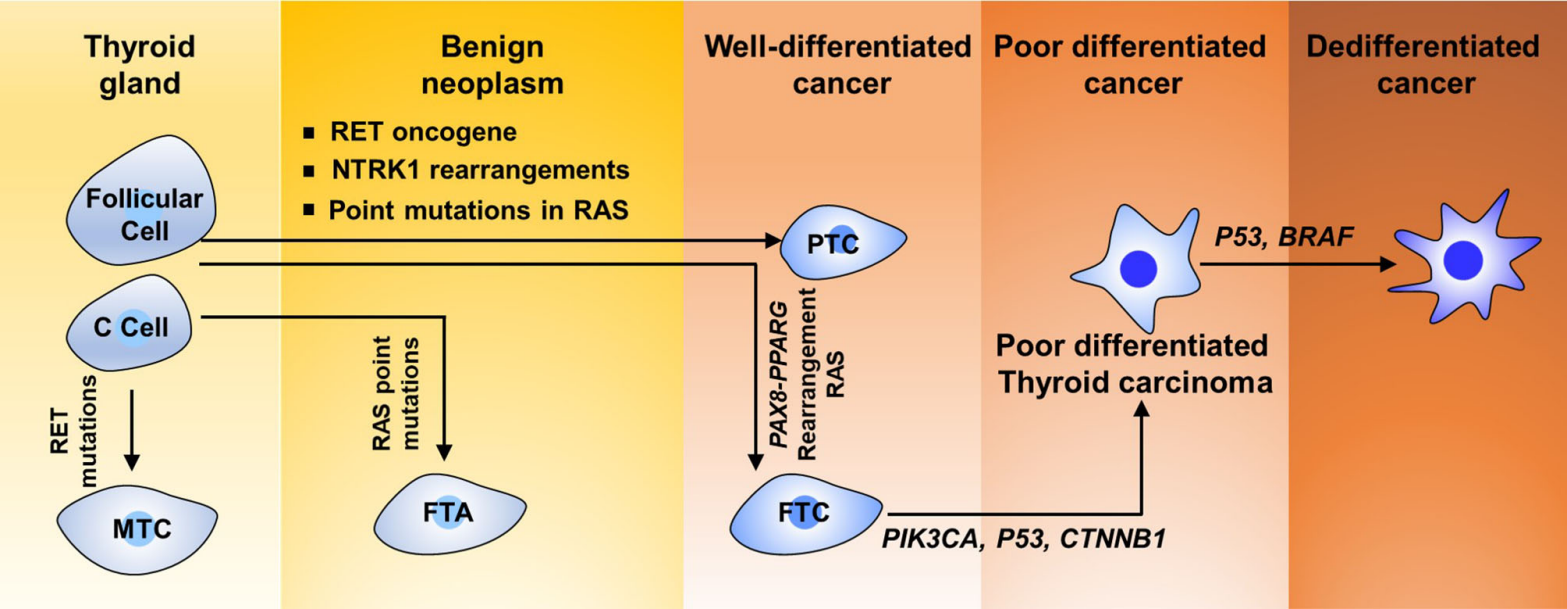
Scheme of the status of dedifferentiation of thyroid carcinoma.

TC includes multiple histological types, such as PTC, follicular thyroid carcinoma (FTC), Hurthle cell carcinoma, poorly differentiated thyroid carcinoma, anaplastic thyroid carcinoma, squamous cell carcinoma, and medullary thyroid carcinoma (**Figure 1**). TC derived from follicular cells could be further divided into three classes based on the diverse histological and pathological elements: well-differentiated TC, poorly differentiated TC, and highly aggressively dedifferentiated TC or ATCs. Well-differentiated TC is composed of PTC and FTC subtypes. Approximately 80% of TCs are diagnosed as PTCs and 10% as FTCs in clinical trials worldwide (22–25). According to surveillance epidemiology and end results (SEER) data from the USA, 5%–10% of TCs originate from calcitonin-producing parafollicular cells (also known as C cells) within the thyroid gland and are known as medullary TC (MTCs) (26–29).

### Papillary Thyroid Carcinoma

Having a papillary architecture and containing carcinoma cells with classic nuclear changes is the unique feature of PTCs (30). PTC incidence is considered to three to six times higher in women than in men among the regions in the world. Exposure to ionizing radiation is considered as an awfully risk element for PTC, as revealed by the higher incidence of PTC among the survivors of the Hiroshima and Nagasaki atomic bombing and among children in Belarus and Ukraine after the Chernobyl accident (31, 32).

### Follicular Thyroid Carcinoma

FTCs are routinely well-differentiated, unifocal, and encapsulated, but they trend toward aggressiveness and extend to blood vessels and invade systemically through the bloodstream. The incidence of FTC is significantly superior in endemic goiter regions, indicating a close correlation between FTC tumorigenesis and iodine deficiency (33–35).

### Anaplastic Thyroid Carcinoma

ATCs are highly undifferentiated and singularly invasive. ATCs are uncommon carcinoma and approximately account for 2%–5% of all TCs (36). ATC cells lack the ability to seize iodine and form thyroglobulin. A large proportion of ATCs originate from pre-existing PTCs and FTCs, but the others seem to derive from *de novo*. The median survival time of ATC patients is only 6 months.

### Medullary Thyroid Carcinoma

MTCs display well-defined carcinoma, which are characterized by lacking a well-formed capsule. High migration ability is the feature of MTCs. MTCs is frequently found to invade outside the thyroid, migrate to lymph nodes and vasculature. Nevertheless, most MTCs are sporadic, and 25% of the incidence of MTCs is hereditary. This familial disease is inherited in an autosomal dominant manner and composed of three subtypes: MEN2A, MEN2B, and familial MTC.

## Genetic Lesions in Thyroid Carcinoma

Like other tumor types, TC tumorigenesis and development require increasing accumulation of diverse genetic and/or epigenetic transformation. Many genetic factors are implicated in TC tumorigenesis. Genetic mutations regulating the biological functions of oncogenic or tumor-suppressive genes have been fully established in thyroid neoplasias. More than one-quarter of MTC cases are hereditary. Approximately 3.5%–6.2% of TCs are idiopathic forms of familial non-MTC (36, 37). In addition, TC develops in patients with cancer syndromes derive from germline mutations (38). Fusion protein generated from rearrangements including NTRK1 has been revealed in 10% of cases of PTCs (39). 1799T>A mutation in BRAF, which results in the switch of V600E at the protein level, was observed in about 40% of PTCs and 20% of ATCs (**Figure 1**) (40). As shown in **Figure 1**, more than 70% of PTCs contain crucial mutations in genes encoding core components of the MAPK signaling pathway, namely, the proto-oncogene RET, which is also known as TRK, RAS, and BRAF (41–43). The *RET* gene encodes a transmembrane receptor protein for neurotrophic growth factor binding and frequent rearrangement between the conserved domain of tyrosine kinase of *RET* and the 5′ region of their partner genes leading to formulate the *RET/PTC* oncogene in about 30% of PTCs (44).

**Figure 2:**
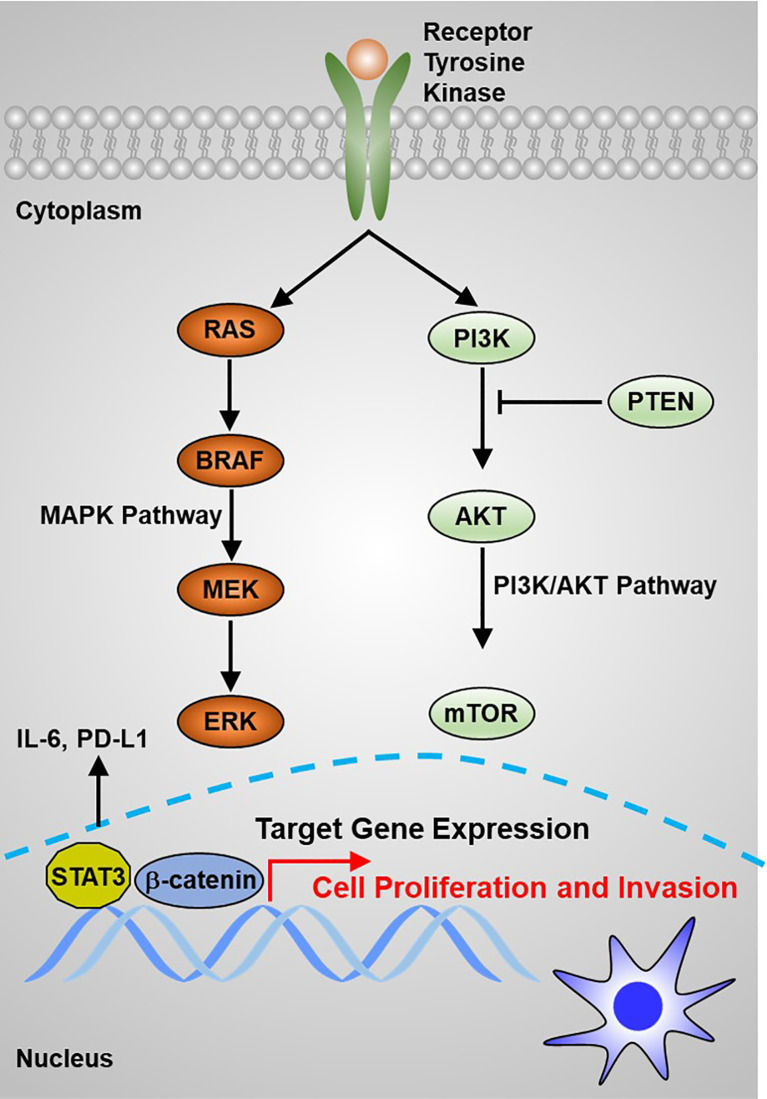
The main signaling pathways that contribute to thyroid carcinoma tumorigenesis are the MAPK and PI3K/AKT pathways.

*RAS* proto-oncogene mutations have been considered to be closely correlated with FTC and have been suggested in about 30% of tumors of the follicular variant of PTC (PTC-FV) (45). Genetic alteration of the *RAS* gene is also the primary molecular incident indicated in poorly differentiated TC (46). Human *RAS* genes, which can be further classified into HRAS, KRAS, and NRAS, encode highly evolutionarily conserved G proteins that reside at the intracellular and transduce signaling transmitted from receptor tyrosine kinases and the downstream signaling along the MAPK and PI3K–AKT (**Figure 2**). Point mutations that activate these signaling pathways typically are codons 12, 13, and 61 of the *RAS* genes (47–49). In TC, codon 61 mutations in NRAS and HRAS are most the frequent. *RAS* mutations are commonly found in various TCs, including 10%–20% of PTCs, 40%–50% of FTCs, and 20%–40% of ATCs (50). Conversely, mutations in MAPK signaling effector genes are correlated with early stages of TC tumorigenesis. Interestingly, mutations in *AKT1* and *BRAF*, but not in *PIK3CA*, have been revealed in poorly differentiated TC (51). Mutations that repress p53 effects are commonly validated in ATCs (**Figure 1**) (52, 53). Abnormal activation of Wnt/β-catenin (54–56) or STAT3 (55, 57, 58) signaling has been commonly observed in TC initiation and progression and predicts poor survival with TC patients. However, other signaling pathways involved in TC initiation and progression are poorly recognized. Thus, a better understanding of the molecular basis and signaling events associated with TC development not only elucidates the mechanism of the TC process but also offers a new window for diagnostic and prognostic prediction and a profound effect on the targeted therapy for patients with TC.

These genetic alterations open new avenues for rational principles of TC tumorigenesis and have been conventionally considered as biomarkers for early diagnosis and prognosis or therapeutic targets for patients with TC. However, increasing studies on the aberrant expression of lncRNA in thyroid neoplasias might contribute to deep elucidation of the genetic basis of TC and shed additional insights into TC diagnosis, prognosis, and therapy.

## Functional Mechanism of lncRNA

LncRNAs are defined as transcripts without coding potential longer than 200 base pairs in length. Like PCGs, lncRNA genomic regions are characterized by the transcriptional start site (TSS) of H3K4 trimethylated abundantly. LncRNA transcripts are composed of several exons that are spliced through canonical mechanisms into a mature transcript and frequently include 5′ caps and 3′ poly(A) tails. However, lncRNAs commonly have fewer exons and are expressed at very weaker levels generally compared with PCGs. Similar to PCGs, lncRNAs are transcribed by RNA polymerase II from independent promoters, but unlike mRNAs, lncRNAs preferentially localize in the nucleus (59, 60), which exert diverse functions, such as regulation of gene expression in *cis* or in *trans*, regulation of splicing, and nucleation of subnuclear domain (61, 62). LncRNAs exert their regulatory effects in regulating signaling transduction and cell fate determination through diverse molecular ways, including interaction with DNA, RNA, and proteins, as well as encoding small peptides (6, 63). Firstly, binding to DNA enables lncRNAs to alter chromatin structure and be involved in the modulation of epigenetic modifications, thereby affecting the expression of target genes. Secondly, lncRNAs function as a molecular sponge to bind to mRNAs or miRNAs, resulting in regulating the stability and translation efficiency of mRNAs or the binding of miRNAs with their own targets.

### MiRNA Sponging

**Figure 3:**
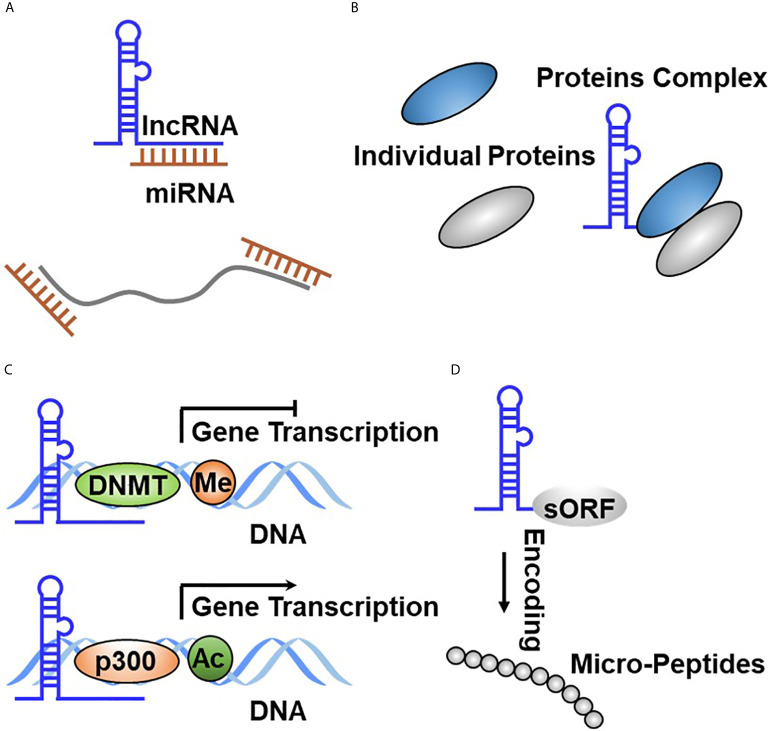
Functional principles of lncRNA in TC. **(A)** LncRNA directly binds to miRNA through complementary base pairing. **(B)** LncRNA binds to protein to form the RNA–protein complex. **(C)** LncRNA regulates gene expression at the transcriptional through binding to DNA through complementary base pairing. **(D)** LncRNA encodes the small peptide.

LncRNA–miRNA interaction displays a common molecular mechanism involved in regulating gene expression and signaling transduction through complementary base pairing (**Figure 3A**). It has been well accepted that miRNAs interact with mRNAs to mediate mRNA turnover, thereby regulating bound mRNA expression (64–66). LncRNAs can interact with both miRNAs and mRNAs, leading to RNA expression regulation being more complicated. In general, lncRNA–mRNA physical interaction affects the degradation and intracellular localization of the target mRNAs, whereas lncRNA–miRNA binding suppresses the competitive interaction of miRNAs binding to their own targets. To date, there are more than 16,000 lncRNAs in the human genome, and some of them were found to exert their functions through acting as miRNA sponge, therefore also known as competitive endogenous RNA (ceRNA) (67–69). LncRNA–miRNA interaction results in the re-expression of target genes inhibited by miRNA. For example, Lei et al. revealed that TUG1 promotes the development of TC cells *via* acting as a ceRNA to sponge miR-145 (70). It is worthy to note that multiple miRNA species might simultaneously bind to a single lncRNA, indicating that the interaction of lncRNAs with other RNA molecules could be more effective and multifunctional (71, 72).

### Protein Binding

The binding of lncRNAs with proteins plays crucial roles in affecting protein stability or subcellular localization, as well as protein complex formation or the release of proteins from their own bound partners, resulting in effecting their biological function. Apart from miRNAs, many lncRNAs have been reported to bind to proteins, thereby regulating their turnover. These binding proteins primarily cover RNA-binding proteins (RBPs), which are routinely accepted as interacting with mRNA (73, 74). Due to the existence of remarkable similarities between lncRNAs and mRNAs, several RBPs have been reported to orchestrate lncRNA functional roles by physical interaction. The binding between lncRNAs and proteins affects protein intracellular localization or degradation (**Figure 3B**). For example, Yuan et al. reported that SLC26A4-AS1 simultaneously binds to DDX5 and the E3 ligase TRIM25, thereby accelerating DDX5 degradation through the ubiquitin–proteasome pathway, resulting in the inhibition of TC cell migration and metastasis (75). Meng et al. defined that LINC00673 physically interacts with EZH2 and DNMT1, which promotes TC progression by inhibiting p53 expression (76). Gou et al. reported that lncRNA AB074169 binds to KHSRP, which decreases KHSRP expression and promotes TC cell proliferation and migration (77).

### DNA Binding and Transcriptional Regulation

Nuclear lncRNAs are diffusely accepted to bind with DNA, including the noncoding or protein-coding region through complementary base pairing. This RNA–DNA interaction resulted in lncRNAs regulating target gene expression in *cis* (lncRNA gene sequence and target gene sequence are in the same chromosome) or in *trans* (lncRNA gene sequence and target gene sequence are in different chromosomes) (**Figure 3C**). A fully studied DNA-interacting lncRNA is XIST, which is involved in regulating the inactivation of X chromosome during early embryonic development (15). To date, a growing body of evidence has reported that lncRNA is documented to bind to DNA. Regrettably, only a subfraction has been functionally determined. FTNRC6C-AS1 was revealed to specifically bind with the promoter of STK4, which significantly promotes STK4 methylation and results in inhibiting STK4 expression in TC (78). LINC00313 was observed to recruit DNMT1 and DNMT3B to bind to the promoter region of ALX4 and significantly promoted methylate ALX4 promoter, resulting in repressing ALX4 expression (79). Pellecchia et al. characterized that PAR5 binds to the promoter of E-cadherin, which relieved negative regulation of E-cadherin by EZH2 (80). LINC00511 was reported to be overexpressed in TC tissues and promoted radiotherapy by binding with TAF1, which activated JAK2 expression at the transcriptional level (81). Xu et al. identified that CCAT1 specifically binds to the promoter of and promotes ASH1L expression in APC and boosts the growth and migration of APC cells (82).

### Encoding Small Peptides

LncRNAs are defined as long transcripts without coding potential. Interestingly, shreds of evidence represented that diverse lncRNAs contain small open reading frames (sORFs) indeed and execute their functions by coding functional peptides (10, 83, 84) (**Figure 3D**). These novel findings broaden the complexity of lncRNA function. The first lncRNA that was identified to have coding potential is ENOD40, which is derived from plants and was found to encode two small peptides that interact with sucrose synthase (85). Subsequently, Rohrig et al. further revealed that the peptide encoded by ENOD40 binds sucrose synthase and elevates the sucrose cleavage activity of sucrose synthase (86). Currently, no lncRNAs that translate into small peptides were identified in TC tumorigenesis and development. The lack of related research may be attributed to several causes. Firstly, the small peptides encoded by lncRNAs are too insecure to monitor. Secondly, a fair portion of these small peptides has no biological function. Therefore, a better understanding is needed to fully recognize the biological function of small peptides encoded by lncRNA in TC initiation and development.

## The Biological Function oF lncRNAs in TC Hallmarks

Different types of cancer share similar hallmarks (87, 88). The crucial roles of lncRNAs in TC initiation and development are evident by the fact that lncRNAs are closely related to TC malignant phenotypes, including sustaining uncontrolled cell growth, having resistance to cellular apoptosis, enhancing invasive ability, and acquiring cancer stem cell-like features. TC-associated lncRNAs facilitate or suppress these malignant behaviors by interacting with their binding macromolecules, indicating rational therapeutic treatment for the therapy of TC.

### Maintaining Proliferative Signaling

**Figure 4:**
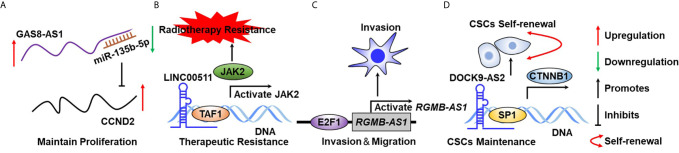
The biological function of lncRNA in TC hallmarks. **(A)** GAS8-AS1 sustains TC cell growth through binding to miR-135b-5p in a complementary base pairing manner. **(B)** LncRNA00511 activates JAK2 expression at the transcriptional level through binding to DNA. **(C)** E2F1 activates RGMB-AS1 expression at the transcriptional level to maintain TC cell migration. **(D)** DOCK9-AS2 binds to SP1 by serving as a scaffold protein to sustain CSC self-renewal. .

Tumor cell uncontrolled proliferation is activated by sustaining proliferative signaling (87, 88). Normal cells restrict cell growth to precisely regulate an appropriate architecture of organisms, whereas transforming cells lead to tumorigenesis by constitutively activating RTK signaling or bypassing cell cycle checkpoints. For example, excessive activation of RTKs has been documented to play essential roles in maintaining TC growth (**Figure 2**). Emerging evidence indicates that lncRNA plays a critical role in supporting TC cell uncontrolled proliferation. For example, Chen et al. reported that lncRNA DGCR5 functions as a novel tumor suppressor in PTC *via* sponging miR-2861 to inhibit PTC cell proliferation and invasion (89). Chen et al. provided evidence that lncRNA GAS8-AS1 represses PTC cell proliferation by the miR-135b-5p/CCND2 signaling axis (90). Pan et al. further proved that GAS8-AS1 holds potential as a novel applicable diagnostic and therapeutic target (91) (**Figure 4A**). Ding et al. proved that SNHG12 promotes cellular proliferation and metastasis of PTC cells through activating the Wnt/β-catenin signaling (92). Duan et al. identified that SNHG3 acts as a new tumor suppressor and attenuates PTC cellular proliferation and metastasis through the AKT/mTOR/ERK signaling pathway (93). Interestingly, another study defined that SNHG3 exerts oncogenic effects in PTC through the miR-214-3p/PSMD10 signaling axis (94), suggesting that the biological function of SNHG3 is controversial. SNHG15 was proved to serve as a ceRNA to sponging miR-200a-3p to activate YAP1–Hippo signaling (95). He et al. identified that NORAD has an oncogenic role to promote PTC tumorigenesis by facilitating cellular proliferation and invasion by sponging and inhibiting miR-202-5p expression (96). Le et al. characterized that WT1-AS promotes PTC cell growth by sponging miR-203 (97). Li et al. identified that SOX2 directly binds to the LINC01510 promoter and promotes its expression at the transcriptional level. LINC01510 executes its function as a ceRNA by specifically binding miR-335, leading to inhibiting miR-335 expressions and accelerating PTC cell growth and migration (98). Liang et al. proved that HCP5 was highly expressed in FTC and promoted the proliferation, invasive ability, and angiogenic capability of FTC cells by acting as a ceRNA sponge for miR-22-3p, miR-186-5p, and miR-216a-5p, which promotes ST6GAL2 (99). Liu et al. identified that lncRNA BANCR promotes TC cell proliferation through activating thyroidal thyrotropin (TSH) receptors and downstream oncogenic signaling (100). Liu et al. recognized that XIST promotes TC cell growth and tumor growth *in vivo* through the miR-34a–MET–PI3K–AKT signaling axis (101). Liu et al. verified that lncRNA UCA1 exerts oncogenic activity by regulating the miR-204/IGFBP5 axis in PTC (102). Meng et al. elucidated that LINC00673 cells dramatically increase the proliferative rate of PTC cells (76). Xia et al. reported that LINC00673 potentiates PTC cell migration and invasion through KLF2 expression (103). Pang et al. defined that targeting of DUXAP8 induces cell apoptosis and attenuates cell proliferation (104). Li et al. reported that TPTE2P1 is required for PTC cell proliferation by sponging and inhibiting miR-520c-3p expression (105). Zhang et al. characterized that BISPR promotes PTC cell proliferation and invasion by depressing miR-21-5p expression (106). Another group further confirmed that NEAT1 increases the resistance effects of ATC cells to cisplatin treatment through sponging miR95p and regulating SPAG9 expression (107). UCA1 attenuates the lactiferous effects exhibited by cytotoxic CD8^+^ T cells on ATC cells *via* the miR-148a/PD-L1 signaling pathway (108).

### Therapeutic Resistance

To date, the therapeutic strategies routinely applied for TC clinical therapy are surgery, radiotherapy, and chemotherapy (109). However, TC cells can gradually develop resistance to therapeutics, particularly chemotherapy, during clinical treatment which can diffusely be attributed to but not limited to the following: 1) considerably enhanced certain transport protein expression that reduces the intracellular concentration of therapeutic drugs, resulting in decreasing chemotherapy efficacy; 2) genetic or epigenetic alterations that enable TC cells to become resistant to cellular death; and 3) augmentation in DNA repair ability (110, 111). Consequently, resistance to chemotherapy is still a major challenge for the clinical therapy of patients with TC because it frequently causes therapeutic failure. Overall, the underlying principles of TC patients resistant to chemotherapeutics remain to be functionally elucidated.

Recently, lncRNAs have emerged as new regulators in chemotherapeutic resistance in TC clinical treatment and have achieved great attention in the field of TC research. Many studies on lncRNA and resistance to therapeutic drugs have been documented over the last decade. For example, Liu et al. identified that MEG3 is weakly expressed in TC tumor tissues, and validated that MEG3 acts as a ceRNA sponge for miR-182, resulting in enhancement of radiotherapy effects (112). Song et al. characterized that GAS5 represses TC cell growth and resistance to doxorubicin treatment in ATC through inhibiting miR-96 expression (113). Another group further confirmed the tumor-suppressor roles of GAS5 in PTC progression (114). Xiang et al. suggested that SLC6A9 was considerably weakly expressed in ^131^I-resistant TC cell lines and overexpression of SLC6A9-5:2 dramatically sensitizes TC cells to ^131^I (115). Chen et al. identified that LINC00511 was markedly overexpressed in TC and silencing LINC00511 promoted the radiosensitivity of TC cells by binding to TAF1 and further promoted JAK2 expression (81) (**Figure 4B**). Xia et al. proved that CCND2-AS1 was significantly highly expressed in PTC and targeting CCND2-AS1 suppressed cell proliferation, migration, and invasion (116). Esposito et al. proved that silencing COMET significantly potentiated the sensitivity of TC cells to vemurafenib, which is an ordinary inhibitor of mutated B-raf (117).

### Activating Migration and Invasion

Tumor metastasis is the real cause of the vast majority of cancer-related deaths (118–120). In brief, the nature of metastasis of TC shares remarkable similarities with other cancer types, whereas epithelial–mesenchymal transition program and increased angiogenesis capability have been considered as critical incidents driving TC metastasis. Many lncRNAs are well established to implicate in the regulation of TC cell invasion and migration, leading to affect TC metastasis. For example, Chen et al. reported that lncRNA TTTY10 enhanced PTC cell migration ability and was defined as novel potential prognostic biomarkers for predicting recurrence of PTC patients (121). Chen et al. revealed that lncRNA ZFAS1 inhibits TC cell proliferation and invasion *via* sponging miR3023p and decreasing cyclin D1 (122). Cui et al. proved that lncRNA-ATB, transcriptionally activated by transforming growth factor beta1 (TGF-β1), was involved in facilitating PTC cell migration and invasion (123). Shen et al. characterized that PROX1-AS1 was overexpressed in PTC and played an important role in PTC proliferation, invasion, and migration (124). NEAT1 was found to be overexpressed in both ATC tissues and cells and was induced in the hypoxia niche. NEAT1 depletion inhibits ATC cell invasion and glycolysis by exerting as a ceRNA sponge of miR-206 and miR-599 under hypoxia (125). HOTAIR was found to promote PTC cell proliferation and invasion *via* implicating the miR-488-5p/NUP205 signaling axis (21, 126). RGMB-AS1 is transcriptionally promoted by E2F1 and activated PTC cell proliferation and invasion (127) (**Figure 4C**). CCAT1 has been reported to promote PTC cell proliferation and migration through inhibiting miR-143 expression (128).

### Sustaining Stem Cell-Like Characteristics

Cancer stem cells (CSCs) are a small subpopulation of cells that reside in tumors, which are well investigated being accounted for tumor initiation, therapeutic resistance, and tumor relapse (129–131). Emerging evidence suggested that Wnt/β-catenin, JAK/STAT, and Hippo/YAP have emerged as crucial players in affecting the stemness of TC CSCs (129, 132). A growing body of evidence validated that lncRNAs participate in CSC formation and maintenance in human cancers, including TC (133, 134). In the past several decades, the biological function of protein-coding genes in TC tumorigenesis and CSC self-renewal maintenance was well established, whereas the roles of lncRNAs have not been fully elucidated. Several pieces of evidence have demonstrated that lncRNA plays a crucial role in sustaining TC CSC self-renewal. For example, Dai et al. proved that lncRNA DOCK9-AS2 interacted with SP1 and activated CTNNB1 expression at the transcriptional level promoting PTC CSC maintenance (135) (**Figure 4D**). LncRNA-H19 accelerates cancer stem-like properties in PTC by activating estrogen receptor β expression (136). PTCSC3 was found to play critical roles in inhibiting PTC stem cell self-renewal (137). In addition, another group further validated that PTCSC3 suppresses PTC cell proliferation and migration by sponging miR-574-5p and represses β-catenin signaling (138).

## Clinical Relevance of lncRNA in TC

Several lncRNAs were identified to be dysregulated in TC and lncRNAs have been proved to hold the potential to function as biomarkers in TC diagnosis and prognosis. **Table 1** summarizes that lncRNAs are dysregulated in TC and have oncogenic and tumor-suppressive roles in TC tumorigenesis and development, given the various regulatory molecular mechanisms and the diverse downstream signaling cascades affected. It is not surprising that several lncRNAs, including many of those emphasized above, have been observed through *in vivo* experiments to be essential players in TC tumorigenesis and progression and display attractive targeted therapeutics (**Table 1**). Given the poor conservation in different species, most of the lncRNA-related studies relied on regulating lncRNA expression in xenograft tumors derived from human cancer cell lines in mice. Similar to miRNAs, lncRNAs have been documented to execute their function either as tumor suppressors or oncogenes or display cellular context-dependent functions (**Table 1**). WT1-AS was inversely associated with the overall survival of patients with TC (97). Overexpression of lncRNA HOTAIR is correlated with worse clinical outcomes in TC (142). Pan et al. defined lncRNA GAS8-AS1 and LPAR4 holds great potential as diagnostic and therapeutic targets (91). Notably, several clinical and analytical factors could influence the final results of the sequencing results (143).

**Table 1:** Dysregulated lncRNAs and their functions in the tumorigenesis of TC.

LncRNAs	Roles in TC	Binding partners	Working models	Significance	Ref.
DGCR5	Tumor suppressor	miR-2861	MiRNA sponge	↓ Proliferation	(89)
GAS8-AS1	Tumor suppressor	miR-135b-5p	MiRNA sponge	↓ Cell cycle	(90, 91, 139)
ZFAS1	Oncogene	miR3023p	MiRNA sponge	↑ Proliferation, invasion	(122)
ATB	Oncogene	TGF-β1	Unknown	↑ Invasion	(123)
DOCK9-AS2	Oncogene	SP1	Transcriptional activation	↑ Stemness, ↑ proliferation	(135)
SNHG12	Oncogene	Wnt/β-catenin	Unknown	↑ Proliferation, invasion	(92)
SNHG3	Tumor suppressor	AKT/mTOR/ERK		↓ Proliferation, invasion	(93)
SNHG15	Oncogene	miR-200a-3p	MiRNA sponge	↑ Proliferation, invasion	(95)
NORAD	Oncogene	miR-202-5p	MiRNA sponge	↑ Proliferation, ↑ invasion	(96)
WT1-AS	Oncogene	miR-203	MiRNA sponge	↓ Proliferation	(97)
H19	Oncogene	miRNA-3126-5p	MiRNA sponge	↑Stemness	(136)
LINC01510	Oncogene	miR-335	MiRNA sponge	↑ Proliferation, invasion	(98)
HCP5	Oncogene	miR-22-3p, miR-186-5p, miR 216a-5p	MiRNA sponge	↑ Proliferation, invasion, ↑ angiogenesis	(99)
UCA1	Oncogene	miR-204	MiRNA sponge	↑ Proliferation, invasion	(102)
AB074169	Oncogene	KHSRP	Protein stability	↑ Proliferation, invasion	(77)
MEG3	Tumor suppressor	miR-182	MiRNA sponge	Radiotherapy	(112)
LINC00673	Oncogene	EZH2, DNMT1	mRNA stabilization	↑ Proliferation	(76, 103)
DUXAP8	Oncogene	miR-20b-5p	MiRNA sponge	↑ Proliferation	(104)
XIST	Oncogene	miR-34a	MiRNA sponge	↑ Proliferation	(101)
GAS5	Tumor suppressor	miR-96	MiRNA sponge	↓ Chemotherapy, proliferation	(113, 114)
NEAT1	Oncogene	miR-206, miR-599	MiRNA sponge	↑ Invasion, ↑ glycolysis, chemotherapy	(107, 125)
HOTAIR	Oncogene			↑ Proliferation, invasion	(126, 140)
CCAT1	Oncogene	miR-143	MiRNA sponge	↑ Proliferation, invasion	(128)
BISPR	Oncogene	miR-21-5p	MiRNA sponge	↑ Proliferation, invasion	(106)
SLC26A4-AS1	Tumor suppressor	DDX5	Protein degradation	↓ Invasion	(75)
LINC00511	Oncogene	TAF1	Transcriptional activation	↑ Radiotherapy	(81)
CCND2-AS1	Oncogene		MiRNA sponge	↑ Proliferation, invasion	(116)
RUNDC3A-AS1	Oncogene	miR-182-5p	MiRNA sponge	↑ Invasion	(141)

### LncRNA as Cancer Biomarkers

Currently, TC diagnosis mainly depends on ultrasonography imaging. In addition, very limited therapeutic alternatives have been applied to TC-related deaths for lacking prognostic biomarkers or therapeutic targets. Thus, therapeutic targets and novel biomarkers are urgently required for developing the clinical therapies of TC. LncRNA can be easily detected from the serum and urine, and many lncRNAs have been reported to secrete from tumor cells into the circulatory system (144). Recent reports have documented that many lncRNAs are stable enough to be detected in the serum or urine of patients with cancer (53). Therefore, examination of the existence of circulating lncRNAs in serum or urine can be employed as diagnostic biomarkers for thyroid cancer diagnosis and prognosis in a noninvasive manner. LncRNA is a useful diagnostic biomarker because dysregulated lncRNA expression is observed across diverse cancers including TC and can be easily detected. Ge et al. identified 795 differentially expressed lncRNAs using the Agilent lncRNA microarray chips approach (145). Teng et al. characterized that eight genes and two novel lncRNAs were identified to correlate with the aggressive and disease-free survival of patients with PTC (146). Song et al. identified 1,878 abnormally expressed lncRNA, of which 1,449 were downregulated and 429 were upregulated, by performing microarray analysis in 86 PTC samples and adjacent noncancerous thyroid tissues (147). Additionally, Yang et al. defined that 751 mRNAs and 675 lncRNAs were dysregulated in the three PTC tissues compared with normal noncancerous thyroid tissues by performing lncRNA microarray analysis (148).

### Diagnostic and Therapeutic Potentials of lncRNA in TC

Many inhibitors have been reported to induce cancer regression in a different type of cancer, yet only a small portion of them have been applied in clinical trials (149–151). Recently, the characterization and application of antisense oligonucleotide (ASO) opens a new avenue for the development of cancer therapeutics, and many of them have been implemented in different clinical trials (152–154). For instance, a phase I study of ASO specifically targeting vascular endothelial growth factor dramatically inhibits TC growth (155). However, no evidence has shown that ASO directly targets lncRNAs to display inhibitory roles in repressing TC, and no ASO targets to lncRNAs have been entered into clinical trials in TC, suggesting that there is an urgent need to define novel ASO directly targeting crucial lncRNAs required for sustaining TC malignant behaviors and to translate them into clinical therapeutic for patients with TC. Prognostically, as mentioned before, many lncRNAs have been reported to be differentially expressed in the progression of TC. For instance, Wen et al. defined that a higher level of ABHD11-AS1 correlated with worse clinical outcomes for PTC patients (156). Pan et al. verified that GAS8-AS1 and LPAR4 hold potential as diagnostics biomarkers or therapeutic targets through characterizing an exome mutational spectrum of PTC patients in a Chinese population (91). Zhou et al. showed that the genetic mutation of GAS8-AS1 could be employed as a diagnosis biomarker for PTC screening (139). Thus, lncRNAs hold great potential to function as novel biomarkers for the early diagnosis and prognosis of patients with TC. Additionally, it is of importance to characterize more dysregulated lncRNAs in TC and translate them into useful biomarkers for diagnostic and prognostic potential of TC patients.

## Conclusions and Perspectives

Numerous studies have demonstrated that lncRNAs can function as regulatory molecules involved in a variety of biological processes. This review highlights the differentially expressed lncRNAs in TC initiation and progression, showing the great potential of lncRNAs as a regulatory molecule, as well as its diagnostic, prognostic, and therapeutic potential in TC studies. LncRNAs have been reported to be involved in various complex functions and molecular mechanisms; however, only a small fraction of lncRNAs and their functions in TC initiation and development have been investigated. Significant improvement has been achieved in uncovering lncRNAs and in understanding their functional roles and molecular mechanism in TC tumorigenesis and development. However, several fundamental issues remain to be addressed so as to obtain a deeper understanding of the biological function and clinical significance of lncRNAs in TC tumorigenesis and development. The functions of the few known lncRNAs still need to be characterized in detail and additional molecular mechanisms of lncRNAs need to be defined. Since many lncRNAs exert diverse functions in affecting multiple cellular processes in many tissues, the elaborate functional principles of lncRNA biological functions require to be understood on a case-by-case basis. In addition, it is indispensable to define whether the sensitivity and reliability of lncRNAs are enough for their substantial clinical application as biomarkers. Whether lncRNAs have advantages compared with other biomarkers used for TC diagnosis needs to be further elucidated. We are optimistic that the application of the new sequencing strategies will decisively determine the functional mechanism of lncRNAs implicated in TC tumorigenesis and development, which will eventually expedite the clinical application of lncRNAs for their use in early diagnosis, clinical therapy, and prognosis evaluation. The vast majority of PTC patients can be managed effectively; however, mortality associated with advanced and iodine-refractory TC remains high. Meanwhile, ATC displays an extraordinary worse prognosis. Whether lncRNAs hold great potential in better understanding the pathogenesis or serve as biomarkers for these advanced TC early diagnosis and effective treatment still needs to be ulteriorly investigated. Epigenetic regulation of lncRNAs bears an exceedingly superb potential in mediating TC initiation and development and should focus on investigating not only through achieving a better understanding of TC but also acquiring therapeutic successes.

Overall, a growing body of evidence demonstrates that specifically targeting lncRNAs might be a novel strategy for the clinical therapy of patients with TC. However, whether lncRNAs could be applied in deeper understanding of the pathogenesis or if it can be potentially used for the biomarkers for TC patients’ early diagnosis and advanced research is needed to further characterize. Additionally, it is of significance to decipher the biological functions of supernumerary lncRNAs correlated with TC tumorigenesis and translate them into clinical biomarkers applied for TC early diagnosis and therapeutic target.

## Author Contributions

QH wrote the manuscript. PL searched the literature and made the figures and tables. JZ was the major contributor in preparing and writing the manuscript. CL, DW, XC, SW, YL, and JW provided help in editing and writing the manuscript. All authors contributed to the article and approved the submitted version.

## Funding

This work was supported by a grant from the Jinan Technology and Innovation Development Scheme (No. 202019024) and National Special Project Funding Project of the Key R&D Program of the Ministry of Science and Technology (No. 2019YFC0119205).

## Conflict of Interest

The authors declare that the research was conducted in the absence of any commercial or financial relationships that could be construed as a potential conflict of interest.

## Publisher’s Note

All claims expressed in this article are solely those of the authors and do not necessarily represent those of their affiliated organizations, or those of the publisher, the editors and the reviewers. Any product that may be evaluated in this article, or claim that may be made by its manufacturer, is not guaranteed or endorsed by the publisher.
